# Evaluation of multidrug-resistant bacteria and their molecular mechanisms found in small animal veterinary practices in Portugal

**DOI:** 10.3389/fcimb.2025.1582411

**Published:** 2025-05-05

**Authors:** Joana Moreira da Silva, Juliana Menezes, Laura Fernandes, Cátia Marques, Sofia Santos Costa, Dorina Timofte, Andreia J. Amaral, Constança Pomba

**Affiliations:** ^1^ CIISA - Centre for Interdisciplinary Research in Animal Health, Faculty of Veterinary Medicine, University of Lisbon, Lisbon, Portugal; ^2^ AL4AnimalS - Associate Laboratory for Animal and Veterinary Sciences, Lisbon, Portugal; ^3^ IMVET- Research in Veterinary Medicine, Faculty of Veterinary Medicine, Lusófona University- Lisbon University Centre, Lisbon, Portugal; ^4^ Animal and Veterinary Research Center (CECAV), Lusófona University, University Centre of Lisbon, Lisbon, Portugal; ^5^ Genevet™, Veterinary Molecular Diagnostic Laboratory, Carnaxide, Portugal; ^6^ Global Health and Tropical Medicine, GHTM, Associate Laboratory in Translation and Innovation Towards Global Health, Associated Laboratory in Translation and Innovation Towards Global Health (LA-REAL), Instituto de Higiene e Medicina Tropical, IHMT, Universidade Nova de Lisboa, Lisbon, Portugal; ^7^ Institute of Infection, Veterinary and Ecological Sciences, School of Veterinary Science, Department of Veterinary Anatomy, Physiology and Pathology, University of Liverpool, Leahurst, United Kingdom; ^8^ Science and Technology School, University of Évora, Évora, Portugal

**Keywords:** small animal veterinary practices, multidrug-resistant organisms, environmental contamination IPC, carbapenem resistance, MRSA, veterinary staff carriage

## Abstract

**Introduction:**

Intensive medical care provided in companion animal practices carries the potential risk of selecting and disseminating multidrug-resistant organisms (MDROs). However, data on infection, prevention and control standards specific to small animal veterinary practices (SAVPs) remains limited. The goal of our work was to evaluate the environmental contamination and staff carriage by MDROs in veterinary practices across Portugal.

**Methods:**

Fourteen SAVPs were enrolled. Environmental samples were collected from critical areas such as operating room, wards and pre-operative area. Veterinary team members voluntarily gave nasal, hand and rectal swabs. All samples were screened for the presence of, including extended-spectrum β-lactamases (ESBL)- and carbapenemase-producing Gram-negative bacteria and methicillin-resistant *Staphylococcus* spp. (MRS). Whole-genome sequencing was performed for carbapenem resistant strains.

**Results:**

Environmental evaluation by surface swabs revealed that 6.5% (n=32/490) were contaminated with multidrug-resistant Gram-negative bacteria. OXA-23-producing *Acinetobacter* spp. (n=5) and IMP-8-producing *Pseudomonas juntendi* (n=2) strains were described on different locations of different SAVPs. Moreover, *Stenotrophomonas maltophilia* (n=12) and *Pseudomonas aeruginosa* (n=3) strains were also found on multiple surfaces of different SAVPs. Three human samples (two rectal, one hand) had carbapenem-resistant *P. aeruginosa* strains by OprD mutations, while *S. maltophilia* strains were recovered from four samples (two rectal, two hands). One nasal swab was positive for carbapenem-resistant *Klebsiella pneumoniae* ST11. Only one SAVP surface was positive for the newly typed for methicillin-resistant *Staphylococcus aureus* (MRSA) ST9220-II. MRSA nasal carriage was found in 14% of samples (n=9/64), with an equal prevalence of ST22-IV and ST8-VI. As for hand samples, MRSA was present in 10.7% (n=4/38), with a predominance of ST8-VI.

**Discussion:**

These emerging data indicate that SAVPs may significantly contribute to the dissemination of MDROs. To address this, rigorous infection, prevention and control (IPC) measures should be implemented, alongside educational workshops directed to all veterinary staff as well as to veterinary and nursing students.

## 1 Introduction

In recent years, antimicrobial resistance has led to approximately 1.27 million global deaths (Murray et al., 2022). The rise in antimicrobial resistance is driven by multiple mechanisms, notably the acquisition of resistance genes, such as *mec*A or *bla*_CTX-M-15_ and *bla*_KPC-like_, through plasmids (Dulon et al., 2011; Mathers et al., 2015). Key culprits include *Escherichia coli*, *Staphylococcus aureus*, *Klebsiella pneumoniae*, *Acinetobacter baumannii* and *Pseudomonas aeruginosa*. These microorganisms are linked to community- and to healthcare-associated infections, posing challenges due to extended treatment periods, increased mortality and higher economic costs. The European Centre for Disease Prevention and Control (ECDC) reported that 4.3 million patients in Europe experienced at least one healthcare-associated infections in 2022-2023 (European Centre for Disease Prevention and Control, 2024). The World Health Organization updated the list of medically important antibiotics, classifying which antibiotics can be used in veterinary medicine, following the prescribing cascade. As expected, last-line antibiotics, such as carbapenems and oxazolidinones, are only to be used in human medicine (European Centre for Disease Prevention and Control, 2024), while 3^rd^ generation cephalosporins, high-priority critically important antimicrobials, are authorized for both human and veterinary medicine (Volkov, 2024). This classification is aligned with European Medicines Agency guidelines, released in 2019 (EMA, 2019).

In the last years, the bond between humans and companion animals has significantly evolved, with animals now being regarded as integral members of the family. This shift has led to an increase not only in the number of veterinary practices, as well as in rapid advancements in small animal care and animals’ lifespan, pressuring veterinarians towards higher standards of healthcare (Eurodev, 2024). Although carbapenems are not approved for veterinary use (EMA, 2019), carbapenem-resistant Enterobacterales, *Acinetobacter* spp. and *P. aeruginosa* are sporadically but consistently reported in companion animals in Europe (Gentilini et al., 2018; Moreira da Silva et al., 2024b). Furthermore, cases of multidrug-resistant organisms (MDROs) such extended-spectrum β-lactamases (ESBL) - producing Enterobacterales and methicillin-resistant *Staphylococcus* spp. (MRS) sharing between humans and companion animals have been reported (Sing et al., 2008; Nienhoff et al., 2009; Grönthal et al., 2018; Hemeg, 2021; Menezes et al., 2022; Menezes et al., 2023), showing the need for a One Health and integrative approach to mitigate the dissemination of MDROs.

The implementation of infection, prevention and control (IPC) programs has long been recognized as a cornerstone to minimize the spread of MDROs within human hospital environments. Not only do they protect hospital staff and patients, IPC also helps to minimize the spillover of resistant bacteria into the community (World Health Organization, 2023). In veterinary medicine, antimicrobial stewardship and IPC programs are rising concepts; notwithstanding, a lack of studies characterizing veterinary IPC programs or which MDROs are circulating within small animal veterinary practices (SAVP) still exists. According to ECDC (European Centre for Disease Prevention and Control, 2016), only genomic-level investigations, such as Whole Genome Sequencing (WGS), can provide the resolution needed to track the distribution of resistance genes across hosts, time, and space. In this way, this study aimed to assess the standard cleanliness of high-touch surfaces in SAVP and to characterize the environmental contamination and staff transient carriage by relevant bacterial pathogens, particularly carbapenem-resistant Gram-negative bacteria and MRS, together with determining bacterial transmission between humans, companion animals, and hospital surfaces through WGS. The outcomes of this study clarified how effective standard IPC protocols in SAVPs are, as well as elucidate on the role SAVPs have in the dissemination of MDROs within a One Health context.

## 2 Materials and methods

### 2.1 Hospitals and clinics characteristics

Fourteen SAVP were enrolled between March 2021 and November 2023, and coded from A to N. SAVP-A to SAVP-G were hospitals and SAVP-H to SAVP-N were clinics. According to the Portuguese legislation, it is mandatory for hospitals to have emergency care available 24/7, as opposed to clinics. SAVPs were located in different regions of Portugal. A previously published self-assessment form was adapted and provided to each SAVP covering different aspects of each facility’s functioning characteristics and IPC practices (Schmidt et al., 2020). After the results evaluation, all SAVP received a written report of the findings and suggestions for improvement.

### 2.2 On-site collection

Contact plates (Plate Count Agar, 28.26 cm²) and surface swabs (TS/5–42 with 10 mL neutralizing buffer, TSC Ltd.) were used to sample critical surfaces in the practice environment. Surface swab samples were collected from a defined area of 100 cm² using a sterile template frame. Both methods were applied to flat surfaces, while only surface swabs were used for irregular surfaces. Samples were taken from the locations as they were found, without any previous cleaning and disinfecting procedure. Surfaces that had been immediately used before sampling were excluded to avoid biased results.

Critical areas for sampling were defined as high-touch and high-rotation surfaces within the practice. Due to their importance for veterinary activities and potential to act as transmission hubs, three locations were always sampled: operating room, wards and pre-operative area. Other locations such as examination rooms and treatment areas were included in the study depending on availability at the time of sampling (if the areas were not in use and were cleaned) and the individual concerns pertaining to each SAVP. **Supplementary Tables 1**, **2** describe the sampling techniques and the surfaces considered in each SAVP.

The disinfection protocols and products in use were defined by each SAVP, and as such, these varied across practices. This information was recorded in the general SAVP self-assessment form previously mentioned.

### 2.3 Human sampling

Sampling was voluntary and written informed consent was obtained. Three swabs (one per nostril and one for both hands) were collected from each member of the working force per SAVP (89 veterinary doctors, 32 nurses, 35 technicians and 13 administrative staff, n=169). Rectal swabs were sampled from 30 people (17.7%, n= 30/169). To reduce potential bias, hand swabs were taken randomly during daily procedures, to avoid unusual hygiene prior to collection. All samples were carefully coded and placed in a cooler until processing. Ethical approval for the study was obtained (CEBEA011/2021) and performed in accordance to relevant guidelines.

### 2.4 Sample analysis

Contact plates were incubated at 37°C and colony forming units (CFU) were counted at 24 and 48 hours of incubation. Following previously established efficacy criteria for aerobic colony count (ACC), a growth >2.5 CFU/cm^2^ indicated failure of the cleaning protocol (Mulvey et al., 2011).

For surface swabs, a cleaning criterion of >1 CFU/cm^2^ was applied (Dancer, 2004) for direct plating growth in non-selective media Brain Heart Agar (Biokar Diagnostics, France) after 24 and 48 hours incubation at 37°C. Following an overnight enrichment step on Brain Heart Infusion broth (Biokar Diagnostics, France) at 37°C, samples were plated onto MacConkey agar supplemented with 1.5 mg/mL cefotaxime or 1.5 mg/mL of meropenem (Thermofisher Scientific, United States) (to select for ESBL-producing or carbapenem-resistant bacteria, respectively), CHROMagar™ *Acinetobacter* supplemented with CHROMagar™ MDR Selective (CR102, Chromagar, Japan), and Brilliance™ MRSA 2 agar (Thermofisher Scientific, United States).

One randomly selected nasal swab was placed overnight on buffered peptone water (Biokar Diagnostics, France) at 37°C, and then plated onto MacConkey agar plates supplemented with 1.5 mg/mL cefotaxime or 1.5 mg/mL meropenem, and on CHROMagar™ *Acinetobacter* supplemented with CHROMagar™ MDR Selective. The other nasal swab was placed overnight on sodium chloride supplemented with 13% tryptone soy broth at 37°C and then plated onto Mannitol Salt Agar (Biokar Diagnostics, France) and Brilliance™ MRSA 2 agar (Thermofisher Scientific, United States).

Rectal and hand swabs were incubated overnight at 37°C in peptone water, followed by plating onto the non-selective and selective media described above.

In all cases, up to three isolates with similar phenotypical appearance were isolated for further characterization. Microdilution antibiotic susceptibility testing was performed for Gram-negative bacteria isolated from MacConkey agar with cefotaxime and meropenem supplementation following EUCAST guidelines (European Committee on Antimicrobial Susceptibility Testing, 2024), to further confirm their resistant phenotype.

### 2.5 Resistance genes detection

DNA was extracted from pure cultures using a boiling extraction method (Dashti et al., 2009). Multiplex PCRs followed by Sanger sequencing were performed as previously described for the detection of β-lactamase genes in Gram-negative bacteria, including ESBL and carbapenemase genes (Menezes et al., 2022). Gram-negative bacteria species identification was performed by sequencing 16S rRNA (Srinivasan et al., 2015).

Staphylococci species identification and *mecA* gene detection was performed as previously described (Couto et al., 2015; Rodrigues et al., 2018). MLST and SCC*mec* identification (Kondo et al., 2007) was performed for methicillin-resistant *Staphylococcus aureus* (MRSA), and one new sequence type (ST) was assigned in accordance to PubMLST (Jolley et al., 2018).

### 2.6 Whole-genome sequencing analysis

One representative resistant strain from each surface was selected for WGS. Genomic DNA was extracted from RNase-treated lysates via NZY Tissue gDNA Isolation kit (NZYTech, Portugal). All libraries for WGS were prepared using TruSeq DNA PCR-Free preparation kit (Illumina, United States). DNA sequencing was performed using Illumina NovaSeq platform with 2×150 bp paired-end reads. *De novo* assembled genomes were obtained using a previously described pipeline (Menezes et al., 2022). ResFinder 4.1 (available at the Centre of Genomic Epidemiology – https://www.genomicepidemiology.org/) and CARD database (available at https://card.mcmaster.ca/home (Alcock et al., 2023),) were used for the screening of the novel generated assemblies for identification of antimicrobial resistance genes. Single-nucleotide polymorphism (SNP) analysis was conducted for each bacterial species using Parsnp v1.2 for multiple sequence alignment of the generated assemblies plus a reference genome. *De novo* assemblies were submitted to NCBI under the bioprojects accession numbers PRJNA1131754 and PRJNA1000421 (OXA-23-producing *Acinetobacter* spp.). Newly identified STs were assigned and submitted to the PubMLST database.

## 3 Results

### 3.1 Surface hygiene evaluation by direct culture

According to contact plate evaluation, 28% of the flat surfaces (n=74/264) failed the cleaning efficacy assessment when interpreted with the criteria of >2.5 CFU/cm^2^, in accordance to Mulvey et al., 2011 (Mulvey et al., 2011) (**Supplementary File 1**). On the other hand, flat and irregular surface evaluation using swabs revealed that 17.7% of surfaces (n=87/490) failed the criteria established by Dancer 2004 of >1 CFU/cm^2^ (Dancer, 2004) (**Supplementary File 1**). Contact plates and swab results were discordant in 27.6% (n=72/264) of flat surfaces. Considering both evaluation methods, flat surfaces cleaning efficacy failure increased to 36.8% (n=96/261). All SAVP had at least one unclean surface (**Supplementary File 1**) with frequencies varying from 3.3% (n=1/30) in SAVP-C to 72% (n=18/25) in SAVP-G.

Surfaces that frequently failed cleaning efficacy assessment included weight scales (60.9%, n=14/23), sinks and taps (44.1%, n=15/34), animal cages (40.5%, n=17/42), shearing blades (41.7%, n=10/24) and keyboards (30.3%, n=10/33).

The average results on the self-assessment form were 37/60 points (25–51 points). The highest scoring practice was SAVP-G; yet, SAVP-G showed the highest level of environmental contamination in the different areas of the hospital, with 72% of surfaces (n=18/25) failing the cleaning efficacy assessment (**Supplementary File 1**).

### 3.2 Surface contamination with Gram-negative bacteria

Considering all surfaces, 6.5% (n=32/490) had positive growth in the selective culture media for Gram-negative bacteria, namely, *Acinetobacter* spp. (3.1%, n=15/490), *Stenotrophomonas* spp. (2.5%, n=12/490), *Pseudomonas* spp. (1.0%, n=5/490), *Enterobacter* spp. (0.4%, n=2/490), *Escherichia* spp. (0.2%, n=1/490), *Klebsiella* spp. (0.2%, n=1/490), and *Leclercia* spp. (0.2%, n=1/490). **Table 1** summarizes all the different bacterial species found on each SAVP.

**Table 1:** Gram-negative bacteria identified upon environmental evaluation of SAVPs A-N.

SAVP	Location	Area	Identified organism	Sequence Type (ST)	NCBI strain ID	Carbapenem Resistance*	
						Imipenem	Meropenem
**C**	Pre-operative area	Plastic mat table 02	*Acinetobacter calcoaceticus-baumannii* complex	NA	NA	≤2 (S)	≤2 (S)
		Plastic mat table 03					
		Weight scale					
		Oxygen balloon					
		Anaesthetic device buttons					
		Tap					
	Wash room	Countertop					
	Operating room 02	Blanket					
**D**	Cat ward	Big cage	*Acinetobacter baumannii*	NA	NA	≤2 (S)	≤2 (S)
		Small cage		NA	NA		
**E**	Pre-operative area	Tap	CTX-M-15-producing *Enterobacter kobei*	NA	NA	≤2 (S)	≤2 (S)
	Cat ward	Cage	CTX-M-15-producing *Escherichia hermannii*	NA	NA	≤2 (S)	≤2 (S)
			*Stenotrophomonas maltophilia*	4	HVD1E4P1	NA	NA
		Cage handle	*Stenotrophomonas maltophilia*	1185 (new ST)	HVD4E4P1	NA	NA
**F**	Wash room	Tap	*Stenotrophomonas maltophilia*	94	EP3G8P3	NA	NA
**G**	Dog ward	Tap	*Pseudomonas aeruginosa*	253	ER3C4P3	>16 (R)	>32 (R)
			CTX-M-3-producing *Klebsiella pneumoniae*	11	ER3C8K2	≤2 (S)	≤2 (S)
	Dog ward	Treatment table plastic mat	IMP-8-producing *Pseudomonas juntendi*	NA	ER1C4P2	16 (R)	32 (R)
		Cage	IMP-8-producing *Pseudomonas juntendi*	NA	ER4C8A1	16 (R)	32 (R)
		Treatment table grids	GES-7-producing *Leclercia adecarboxylata*	NA	ER5C2K1	≤2 (S)	≤2 (S)
	Isolation unit	Cage	*Stenotrophomonas maltophilia*	120	ER3F8A1	NA	NA
		Plastic mat on countertop	*Stenotrophomonas maltophilia*	115	ER2F4P2		
			*Pseudomonas aeruginosa*	253	ER2F8P2	16 (R)	>32 (R)
	Cat ward	Tap	*Stenotrophomonas maltophilia*	120	ER3D8P2	NA	NA
			CTX-M-15- producing *Enterobacter hormaechei*	NA	ER3D2K1	≤2 (S)	≤2 (S)
**H**	Treatment area	Weight scale	OXA-23-producing *Acinetobacter lwoffii*	NA	B4Z4A1 B4Z8A1	8 (R)	4 (S)
	Treatment room	Stainless steel supporting tray	OXA-23-producing *Acinetobacter lwoffii*	NA	B11Z4A1	8 (R)	8 (R)
		Keyboard computer	OXA-23-producing *Acinetobacter lwoffii*	NA	B12Z8A1	4 (I)	4 (S)
	Waiting room	Weight scale	OXA-23-producing *Acinetobacter schindleri*	NA	B1E8A1	8 (R)	4 (S)
**I**	Cat examination room	Sink	*Pseudomonas aeruginosa*	267	A6E4P2	8 (R)	4 (I)
**K**	Dog examination room	Sink	*Stenotrophomonas maltophilia*	967 (new type)	C1DE4P2	NA	NA
	Cat examination room	Sink	*Stenotrophomonas maltophilia*	5	C6EE4A1	NA	NA
**L**	Examination room 01	Sink	*Stenotrophomonas maltophilia*	39	B4EE4A2	NA	NA
**N**	Recovery ward	Cage	*Stenotrophomonas maltophilia*	27	EX2C4P3	NA	NA
	Fomites	Practice’s mobile phone	*Stenotrophomonas maltophilia*	115	EX3I8A1	NA	NA
		Hand cloth	*Stenotrophomonas maltophilia*	115	EX2I4A1	NA	NA

NA, Not applicable; S, Susceptible; I, Susceptible to increased exposure; R, Resistant.

*EUCAST 2024 breakpoints (European Committee on Antimicrobial Susceptibility Testing, 2024) – for Enterobacterales, breakpoints are as follows: imipenem (S ≤2; R > 4) and meropenem (S ≤2; R > 4); for *Acinetobacter* spp., imipenem (S ≤2; R > 4) and meropenem (S ≤2; R > 2); and for *Pseudomonas* spp., imipenem (S ≤0.001; R > 4) and meropenem (S ≤2; R > 8). There are no breakpoints for *S. maltophilia* due to their naturally occurring carbapenemases, making them intrinsically resistant.

Carbapenem-resistant non-fermenting Gram-negative bacteria were also recovered on 58.9% (n=20/32) of contaminated surfaces. Overall, 57.1% (n=8/14) of SAVP had at least one positive surface for carbapenem-resistant Gram-negative bacteria – three hospitals and five clinics (**Table 1**).

Considering veterinary hospitals, *Stenotrophomonas maltophilia*, which is intrinsically resistant to multiple classes of antibiotics, including carbapenems (due to intrinsic carbapenemases L1 and L2 (Brooke, 2021)), was identified on two surfaces of the cat ward of SAVP-E, and on the tap of the washing area of SAVP-F. In SAVP-G, *P. aeruginosa* and *S*. *maltophilia* were present on different surfaces (**Table 1**). Additionally, carbapenem-resistant *Pseudomonas juntendi* was found on a plastic mat on the countertop and inside one dog cage (**Table 1**).

Regarding veterinary clinics, carbapenem-resistant *Acinetobacter* spp. was found on four surfaces of SAVP-H. Carbapenem-resistant *P. aeruginosa* (A6E4P2) was found on the cat examination room sink of SAVP-I. *S. maltophilia* were found on the examination room sinks of SAVP-K) and SAVPL. For SAVP-N, it was present, on the post-surgery cage the practice’s mobile phone and a hand cloth (**Table 1**).

All *S. maltophilia* were susceptible to trimethoprim/sulfamethoxazole (MIC < 0,001 mg/L). All carbapenem-resistant isolates were characterized using WGS.

#### 3.2.1 Whole-genome sequence analysis of carbapenem-resistant Gram-negative bacteria from surfaces

The two *S. maltophilia* isolated from SAVP-E belonged to different STs, namely, ST4 in a cat cage (HVD1E4P1), and the newly assigned ST1185 on a cage handle from the same cat ward (HVD4E4P1). The *S. maltophilia* from SAVP-F belonged to ST94 (EP3G8P3).

On SAVP-H, all the carbapenem-resistant *Acinetobacter* spp. (n=5) harbored *bla*_OXA-23_ carbapenemase gene in the same plasmid across different sub-species, as previously described by our group (Moreira da Silva et al., 2024a).

The two *P. aeruginosa* strains from SAVP-G belonged to ST253 and were unrelated (single nucleotide polymorphism (SNP) difference > 14, **Supplementary Table 4**) (Schürch et al., 2018). Both strains were carbapenem-resistant, having the same mutations on OprD porin channel, and on *nalC* and *mexR* genes (MexAB-OprM efflux pump repressor and regulator, respectively) (**Table 2**). Both *P. juntendi* were IMP-8-producers (ER1C4P2 and ER4C8A1) and were considered unrelated by using the SNP relatedness cut-off of *P. aeruginosa* (SNP difference >14, **Supplementary Table 5**), as no cut-of has been proposed for this sub-species (Schürch et al., 2018). The *bla*_IMP-8_ carbapenemase gene was possibly chromosomally inserted as no plasmids were detected (Aytan-Aktug et al., 2022).

**Table 2:** Mutations on OprD porin channel and MexAB-OprM efflux pump of *P. aeruginosa* strains (n=8).

SAVP	Type of Sample	NCBI strain ID	ST	Serotype	OprD	MexAB-OprM	Carbapenem resistance*	
							Imipenem	Meropenem
**G**	Environmental	ER3C4P3	253	O10	T103S; K115T; F170L; E185Q; P186G; V189T; R310E; A315G; A425G	*nalC* (G71E; A145V; S209R); *mexR* (V126E)	>16 (R)	>32 (R)
	Environmental	ER2F8P2	253	O10	T103S; K115T; F170L; E185Q; P186G; V189T; R310E; A315G; A425G	*nalC* (G71E; A145V; S209R); *mexR* (V126E)	>16 (R)	>32 (R)
**I**	Environmental	A6E4P2	267	O2	S278P	Wild-type	>8 (R)	4 (I)
**E**	Rectal swab	HVD5R4P1	27	O1	Stop Codon (TAG) at position 94	*nalC* (G71E; S209R); *mexR* (V126E; V132A)	>16 (R)	>32 (R)
**I**	Rectal swab	A5R4P1	244	O2	Wild-type	Wild-type	≤1 (S)	≤1 (S)
**K**	Rectal swab	C8R8P1	274	O3	D43N; S57E; S59R; E202Q; I210A; E230K; S240T; N262T; A267S; S278P; A281G; K296Q; Q301E; R310G; V359L; M372V; S373D; D374S; N375S; N376S; V377S; G378S; K380A; N381G; Y382L	*nalC* (S209R, G71E)	>8 (R)	8 (R)
**I**	Nasal swab	A6Np4P1	244	O2	Wild-type	Wild-type	≤1 (S)	≤1 (S)
**K**	Hand swab	C11Hp4P1	274	O3	Stop Codon (TAG) at position 378; filled with deletions	*nalC* (G71E,S209R)	>16 (R)	>32 (R)

S, Susceptible; I, Susceptible to increased exposure; R, Resistant. *EUCAST 2024 breakpoints (European Committee on Antimicrobial Susceptibility Testing, 2024) –for *Pseudomonas* spp., imipenem (S ≤0.001; R > 4) and meropenem (S ≤2; R > 8).

Finally, one *S. maltophilia* ST115 strain (ER2F4P2) and two ST120 strains (ER3F8A1 and ER3D8P2) were identified. Interestingly, the two ST120 were closely related (5 SNP difference) showing its likely dissemination between the isolation unit and cat ward of SAVP-G (**Supplementary Table 6**).

SAVP-I, SAVP-K and SAVP-L are veterinary clinics from the same business group (i.e. some staff alternate between veterinary practices); however, none of the isolated strains were shared between practices. The SAVP-I carbapenem-resistant *P. aeruginosa* ST267 strain (A6E4P2) had one mutation on OprD porin channel and a wild-type MexAb-OprM efflux pump (**Table 2**). As for *S. maltophilia* strains, multiple STs were identified, namely ST5 (C6EE4A1) and a newly assigned ST967 (C1DE8A3) in SAVP-K; and ST39 strain (B4EE4A2) in SAVP-L.

Lastly, on SAVP-N, there was one *S. maltophilia* ST27 strain (EX2C4P3) and two ST115 strain (EX3I8A1 and EX2I4A1; 10 SNP difference).

### 3.3 Carbapenem-resistant Gram-negative bacteria transient carriage by veterinary staff

Diverse and normal microbial flora was observed across the 30 fecal samples available. Carbapenem-resistant non-fermenting Gram-negative bacteria were isolated from 16.7% (n=5/30) participants, including *P. aeruginosa* (n=3) and *S. maltophilia* (n=2).

The three *P. aeruginosa* strains were isolated from participants of different SAVP, namely a ST27 (HVD5R4P1) in one veterinarian from SAVP-E; a ST244 (A5R4P1) in one veterinarian from SAVP-I; and a ST274 (C8R8P1) in one nurse from SAVP-K.

*P. aeruginosa* ST244 strain was carbapenem-susceptible and was found to have a wild-type OprD porin channel and MexAB-OprM efflux pump. *P. aeruginosa* ST27 strain had an early stop codon on *oprD* together with mutations on *nalC* and *mexR; and P. aeruginosa* ST274 had several mutations on *oprD*, thus explaining their carbapenem-resistant phenotype (**Table 2**).

One *S. maltophilia* ST317 strain (B3R4P1) was detected in a nurse from SAVP-L, and one *S. maltophilia* ST84 strain (C10R8A1) in a technician from SAVP-K, both susceptible to trimethoprim/sulfamethoxazole.

Gram-negatives were rarely isolated from nasal swabs (1.2%, n=2/169) using selective media. One veterinarian from SAVP-G was positive for an imipenem-resistant CTX-M-3-producing *K. pneumoniae* ST11 (R11Np4K1; MIC= 8 mg/mL), albeit susceptible to meropenem, showing a 15% truncated Omp36K in the WGS analysis (**Supplementary Figure 1**). One veterinarian from SAVP-I was positive for a carbapenem-susceptible *P. aeruginosa* ST244 (A6Np4P1) that was related to the ST244 (A5R4P1, SNP difference ≤ 3, **Supplementary Table 3**) isolated from the rectal swab of a distinct veterinarian from the same veterinary practice.

Only 1.8% (n=3/169) hand swabs were positive for Gram-negative bacteria. One *P. aeruginosa* ST274 strain (C11Hp4P1) was found on a technician from SAVP-K, showing an early stop codon on *oprD*, leading to failure in of the protein expression; and mutations on *nalC* (**Table 2**). In SAVP-N, two related *S. maltophilia* ST115 (6 SNP difference, **Supplementary Table 7**) were detected on the hands of a veterinarian (X4Hp4P2) and a technician (X3Hp4P1). These were closely related to the strains present in the fomites (≤ 10 SNPs difference, **Supplementary Table 7**), showing likely dissemination across the practice fomites and staff.

### 3.4 Surface contamination with methicillin-resistant *Staphylococcus* spp.

Nearly five percent (n=23/489) of surfaces were contaminated with methicillin-resistant *Staphylococcus* spp. (MRS). Two veterinary hospitals (SAVP-A; and SAVP-C) and one clinic (SAVP-N) had surfaces contaminated with MR *Staphylococcus pseudintermedius* (MRSP) (n=4) (**Table 3**). Overall, coagulase-negative MRS isolates predominated (78.3%, n=18/23), especially MR *Staphylococcus epidermidis* (72%, n=13/18) (**Table 3**). One newly typed ST9220 (clonal complex, or CC, 5) MRSA, harboring SCC*mec*II, was isolated from a cage at the cat ward of hospital SAVP-E.

**Table 3:** Methicillin resistant *Staphylococcus* spp. on environmental evaluation of SAVPs A-N.

SAVP	Location	Area	Identified organism
**A**	Waiting room	Weight scale	MRSP
	Operating room	Stainless steel supporting tray	MRSE
**B**	Treatment area	Treatment table 02	MRSE
		Computer keyboard	
	Cat ward	Cage 06 (group on the left)	MRS
	Operating room 02	Computer keyboard	MRSE
**C**	Pre-operative area	Tap	MRSP
		Anaesthetic device buttons	
**H**	Ultrasound room	Ultrasound keyboard	MRSE
**J**	Examination room/Treatment room	Computer keyboard	MRS
	Examination room/Treatment room	Desk	MRSE
**K**	Pre-operative/Treatment area	Fridge handle	MRSE
	Operating room	Anesthesia tent - Outside	MRSE
		Operating table - Head	MRSE
		Blanket	MR *Staphylococcus hominis*
	Ward	Inside knob	MRSE
		Drawer handles	
		Pink blanket	
		Tap	
		Shearing blade	*MR Staphylococcus warneri*
**E**	Operating room	Detergent dispenser	MRS
	Cat ward	Cage left top	MRSA
**N**	Others	Keyboard personal computer	MRSP

MRSA, Methicillin resistant *S. aureus*; MRSE, Methicillin resistant *S. epidermidis*; MRS, Methicillin resistant *Staphylococcus* spp.; MRSP, Methicillin resistant *S. pseudintermedius.*

### 3.5 Methicillin-resistant *Staphylococcus* spp. transient carriage by staff

A total of 169 nasal swabs were collected, of which 38% were positive for MRS (n=64/169). Amongst these, 14% were MRSA (n=9/64). Further characterization identified them as belonging to different CCs: ST22-IV (also known as EMRSA-15) (n=2) (SAVP-A, n=1; SAVP-B, n=1) and ST974-IV (SAVP-I, n=1) from CC22; ST8-VI (n=3) (SAVP-B, n=2; SAVP-L, n=1) from CC8; ST30-V (SAVP-C, n=1) from CC30; ST9220-II (SAVP-E, n=1) and ST125-IV (SAVP-F, n=1) from CC5. MRSP was not detected.

Considering hand swabs, 22.4% (n=38/169) were positive for MRS, and again, none was identified as MRSP. MLST and SSC*mec* cassette characterization of MRSA (10.5%, n=4/38) yielded the following classifications: ST8-VI (n=2) (SAVP-I, n=1; SAVP-L, n=1) from CC8, ST9220-II (SAVP-E, n=1) from CC5 and non-typable ST from CC45 (SAVP-B, n=1) – it was not possible to type this strain of MRSA, albeit it carried SCC*mec*V.

Both MRSA ST8-VI strains belong to different clinics, despite being part of the same business group, with these members rotating between the different practices.

Interestingly, the new MRSA ST9220-II was only identified on SAVP-E in one surface, one nasal swab from a nurse, and one hand swab from a technician, pointing to an ongoing dissemination within this veterinary hospital.

## 4 Discussion

This study depicts the environmental contamination and veterinary staff carriage by resistant bacteria towards medically important antimicrobials within veterinary clinics and hospitals in Portugal. It was found that 6.5% (n=32/490) surfaces analyzed were positive for MDROs, amongst which were detected carbapenem-resistant bacteria, namely OXA-23-producing *Acinetobacter* spp., (n=5) and IMP-8 *P. juntendi* (n=2). Veterinary carriage analysis revealed that 38% and 22.4% were positive for MRS in their nasal cavities and hand swabs, respectively. For rectal swabs, only 16.7% yielded carbapenem-resistant bacteria carriage.

Notably, carbapenem-resistant Gram-negative bacteria (*Acinetobacter* spp., *Pseudomonas* spp. and *S. maltophilia*) and MRSA, belonging to epidemiologically relevant clones such as EMRSA-15, were found in several high-touch surfaces and fomites of SAVPs. The SAVP-G is a particularly interesting case, since, despite having a high self-assessment score about ongoing IPC procedures, it showed the highest level of environmental contamination, together with the isolation of the highest diversity of resistant bacteria. These results highlight that inadequate/insufficient IPC programs or the lack of compliance to them, may facilitate environmental contamination by MDROs, increasing the chance of their dissemination within SAVP and into the environment and community (e.g., staff, tutors, animal patients).

The hygiene evaluation of high-touch surfaces was conducted by two distinct methods, using contact plates and/or surface swabs. The observed discrepancies between results could be attributed to the design of the contact plates, which support the growth of all types of microorganisms (such as fungi and yeast), while surface swabs are loaded with a universal neutralizing liquid buffer specifically geared towards bacterial growth. Conversely, swabs can be used to evaluate irregular surfaces, supporting the use of both techniques for a more complete evaluation of environmental contamination. Considering both methods, 96 surfaces failed the cleaning efficacy assessment (Dancer, 2004). Coincidently, most of these were high-rotation surfaces such as weight scales, sinks and taps, animal cages and computer keyboards.

The worldwide ongoing rising in carbapenem-resistance is worrisome, as these are last-line antibiotics (EMA, 2019). In this study carbapenem-resistant bacteria and possible dissemination events within some SAPV were detected. On SAVP-H, OXA-23-producing *Acinetobacter* spp. strains were found on different surfaces of the same room, indicating a possible transfer (previously published (Moreira da Silva et al., 2024a)).

The detection of various clones of carbapenem-resistant *P. aeruginosa* strains on high-touch surfaces is worrisome. Between 2022-2023, 7.9% of healthcare-associated infections reported in Europe in the human healthcare setting were caused by *P. aeruginosa*, of which 29.7% were resistant to carbapenems (European Centre for Disease Prevention and Control, 2024). *P. aeruginosa* ST244, ST27 and ST253 clonal lineages are commonly associated with outbreaks and nosocomial infections in humans (del Barrio-Tofiño et al., 2020). A previous study conducted in a veterinary teaching hospital reported the presence of *P. aeruginosa* ST244 on sinks (Soonthornsit et al., 2023), while *P. aeruginosa* ST27 has higher affinity towards cystic-fibrosis patients (Weimann et al., 2024). There aren’t any guidelines that support rectal sampling on healthy healthcare-workers in human medicine, as studies have shown that in low prevalence settings of MDROs, the transient carriage by healthcare-workers will also be low (Bassyouni et al., 2015; Decker et al., 2018). Since in this study’s context, the reality of MDROs carriage by healthcare-workers is unknown, we decided to evaluate it. The low percentage of rectal samples positive for carbapenem-resistant bacteria (n=5/30, 16.7%) of veterinary healthcare-workers aligned with what is already known in human medicine as they work in a low-exposure setting to MDROs.

Strains reported on our study were from healthy human samples (*P. aeruginosa* ST244 from one nasal and one rectal swab from unrelated individuals; carbapenem-resistant *P. aeruginosa* ST27 from one rectal swab). Although SAVP-I and SAVP-K belong to the same business group, it was interesting to perceive that each SAVP had its own associated clone shared between staff members, namely, *P. aeruginosa* ST244 on SAVP-I, and carbapenem-resistant *P. aeruginosa* ST274 on SAVP-K (rectal swab and hand swab from unrelated individuals). Although none of these clones were recovered from the environment, their sharing among team members points to their potential for dissemination. These results highlight the importance of screening veterinary staff as part of an effective IPC protocol to identify potential carriers, thereby preventing transmission to patients, staff, and the community. Moreover, a carbapenem-resistant *P. aeruginosa* ST253 was present on a plastic mat on a countertop inside an isolation unit (SAVP-G), an area with specific cleaning and disinfection protocols (Weese, 2004), and although it is not expected for this area to be completely sterile, potentially nosocomial microorganisms should not be present.

Genetically unrelated IMP-8-producing *P. juntendi* strains were present on the inside of an empty cage and on the plastic cover of the treatment table in the dog ward of SAVP-G. To the best of our knowledge, this is the first description of IMP-8-producing *P. juntendi*. So far, only one description of carbapenem-resistant *P. juntendi*, harboring an IMP-1, has been made in a Chinese human patient (Zheng et al., 2022). This data highlights the capacity of this species to acquire and disseminate resistance genetic elements, making it a pathogen to be considered in epidemiological surveillance schemes.

*S. maltophilia* is an opportunistic pathogen with intrinsic resistance to many antibiotics, including carbapenem, posing major challenges in clinical settings (Mojica et al., 2022). In our study, multiple clones (including the new ST967 and ST1185) of *S. maltophilia* were described in the environment of various SAVPs, as well as colonizing staff members. These bacteria are ubiquitous in the environment, yet they have also been associated with nosocomial and community-acquired infections (Gröschel et al., 2020; Mojica et al., 2022; European Centre for Disease Prevention and Control, 2024) The relatedness between environmental and pathogenic *S. maltophilia* strains has been described, indicating that the environment may be a source of human contamination –including from sinks and taps (Mojica et al., 2022). As expected, the majority of the contaminated surfaces in our study were water-related. The spread of *S. maltophilia* through fomites ultimately causing human infection has also been described (Gideskog et al., 2020). The occurrence of *S. maltophilia* ST115 strains on the handcloth, the practice’s mobile phone and on the hands of two staff members of SAVP-N demonstrate that such objects likely acted as fomite and contributed to dissemination of this clone (Gideskog et al., 2020). Yet, in veterinary medicine, reports of infections caused by this agent are rare. Nonetheless, *S. maltophilia* ST115 clonal lineage has been associated with infections in cats (Shimizu et al., 2021), underscoring its disease-causing capability and the importance of closely monitoring it.

In Europe and according to the 2022–2023 ECDC report, 23.7% of *S. aureus* causing healthcare-associated infections were MRSA, showing a 6.3% decrease from the previous report (European Centre for Disease Prevention and Control, 2024). EMRSA-15 is a major clone found in hospitals and in the community in Portugal (Tavares et al., 2013). This epidemic clone has also been described on clinical strains from pets (Couto et al., 2015; Costa et al., 2022), with studies showing that working in close contact with companion animals is a risk factor for MRSA carriage (Weiß et al., 2013; Bal et al., 2016; Feßler et al., 2018; Rodrigues et al., 2018). The newly described MRSA ST9220-II, belonging to CC5, was found on SAVP-E. The presence on a surface as well as on a nasal swab from a nurse and a hand swab from a technician suggests that this clone might be spreading within this veterinary practice, possibly through contaminated surfaces.

A Portuguese study identified 61% of nasal carriage of MRS among veterinary professionals (Rodrigues et al., 2018), which is higher than what was found in the present study. However, this same study reported 14% carriage of MRSA, comparable to our findings, with EMRSA-15 also being the most prevalent clone (Rodrigues et al., 2018). The frequent colonization by MRS and MRSA reported in these two studies showcases that veterinary healthcare providers may contribute to the transmission cycle of these pathogens into the community. The frequency of MRSA recovered from hand swabs was lower. It is known that hand hygiene is a pivotal measure in IPC programs, with studies showing that improvements in the healthcare workers’ hand hygiene protocols cause a direct decrease in the carriage of MRSA (Marimuthu et al., 2014).

Overall, the detection of MRS and carbapenem-resistant Gram-negative bacteria on high-touch surfaces in SAVPs underscores the need for strict IPC procedures. These measures are essential not only to protect patients but also to address the ongoing antimicrobial resistance crisis.

## 5 Conclusion

The current study depicts varying levels of environmental contamination and staff carriage of carbapenem-resistant Gram-negative and MRS strains in SAVP across Portugal. These findings question the effectiveness of ongoing IPC protocols and highlight the risk of environment/staff cross-contamination through high-touch surfaces. This data suggests that SAVP may play an active role in the spread of priority pathogens resistant to medically important antimicrobials, emphasizing the need for targeted educational workshops for veterinary healthcare students and professionals. In the long run, implementing and monitoring evidence-based IPC protocols and staff training should be mandatory to ensure strict compliance in SAVP.

## Data Availability

The datasets presented in this study can be found in online repositories. The names of the repository/repositories and accession number(s) can be found below: https://www.ncbi.nlm.nih.gov/, PRJNA1131754 https://www.ncbi.nlm.nih.gov/, PRJNA1000421.
