# Supplementary material for: Evaluation of multidrug-resistant bacteria and their molecular mechanisms found in small animal veterinary practices in Portugal
Source: Front Cell Infect Microbiol. 2025 May 5;15:1582411. doi: 10.3389/fcimb.2025.1582411 (PMC12086164; doi:10.3389/fcimb.2025.1582411)
Supplement: Supplementary file 1 [file Table1.docx]

Supplementary Material

Supplementary Table 1 – Environmental sampling sites in hospitals A-G

| **Date of collection** | **Small animal veterinary practices** | **Areas** | **Specific areas** | **Sampling methods** |
| --- | --- | --- | --- | --- |
| March 2021 | SAVP-A | Operating room | Anaesthetic device | SS |
|  |  |  | Ultrasound keyboard | SS |
|  |  |  | Stainless steel supporting table | CP+SS |
|  |  |  | Operating table | CP+SS |
|  |  | Cat ward | Small cage | CP+SS |
|  |  |  | Large cage | CP+SS |
|  |  |  | Computer keyboard | SS |
|  |  | Examination room 01 | Examination table | CP+SS |
|  |  | Examination room 02 | Examination table | CP+SS |
|  |  | Locker room | WC latch | SS |
|  |  |  | WC cistern | SS |
|  |  | Waiting room | Weight scale | CP+SS |
| April 2021 | SAVP-B | Operating room 01 | Operating table | CP+SS |
|  |  | Operating room 02 | Operating table | CP+SS |
|  |  |  | X-ray table | CP+SS |
|  |  |  | Ultrasound keyboard | SS |
|  |  |  | Ultrasound table | SS |
|  |  | Treatment area | Treatment table 01 | CP+SS |
|  |  |  | Treatment table 02 | CP+SS |
|  |  |  | Computer keyboard | SS |
|  |  | Examination room 02 | Examination table | CP+SS |
|  |  | Examination room 03 | Examination table | CP+SS |
|  |  | Locker room | WC latch | SS |
|  |  | Laboratory | Microscopy countertop | CP+SS |
| February 2022 | SAVP-C | Operating room 01 | Operating table - Head | CP+SS |
|  |  |  | Operating table - Toes | CP+SS |
|  |  |  | Anaesthetic device keyboard | SS |
|  |  |  | Anaesthetic device | CP+SS |
|  |  |  | Vital sign monitor buttons | SS |
|  |  |  | Anaesthetic device buttons | SS |
|  |  |  | Oxygen balloon | SS |
|  |  |  | Peroxide hydrogen machine | SS |
| February 2022 | SAVP-C | Operating room 01 | Stainless steel supporting tray | SS |
|  |  | Operating room 02 | Operating table -Head | CP+SS |
|  |  |  | Operating table - Toes | CP+SS |
|  |  |  | Blanket | CP+SS |
|  |  |  | Anaesthetic device | CP+SS |
|  |  |  | Anaesthetic device buttons | SS |
|  |  |  | Microscope cover | SS |
|  |  |  | Buttons on respiratory device | SS |
|  |  |  | Stainless steel supporting tray | CP+SS |
|  |  | Pre-operative area | Plastic mat table 01 | SS |
|  |  |  | Plastic mat table 02 | SS |
|  |  |  | Table grids 01 | SS |
|  |  |  | Table grids 02 | SS |
|  |  |  | Plastic mat table 03 | SS |
|  |  |  | Tap | SS |
|  |  |  | Weight scale | CP+SS |
|  |  |  | Oxygen balloon | SS |
|  |  |  | Anaesthetic device buttons | SS |
|  |  |  | Disinfectant dispenser | SS |
|  |  |  | Shearing blade | CP+SS |
|  |  |  | Soap dispenser | SS |
|  |  | Wash room | Countertop | CP+SS |
| April 2022 | SAVP-B | Operating room 02 | Stainless steel supporting tray 01 | CP+SS |
|  |  |  | Stainless steel supporting tray 02 | CP+SS |
|  |  |  | Stainless steel supporting tray 03 | CP+SS |
|  |  |  | Oxygen balloon | SS |
|  |  |  | Anaesthetic device | SS |
|  |  |  | Operating table | CP+SS |
|  |  | Cat ward | Cage 06 | CP+SS |
|  |  |  | Cage 08 | CP+SS |
|  |  |  | Cage 06 (group on the left) | CP+SS |
| April 2022 | SAVP-B | Cat ward | Infusion pump | SS |
|  |  | Ultrasound room | Ultrasound table | CP+SS |
|  |  |  | Ultrasound bed | CP+SS |
|  |  |  | Ultrasound keyboard | SS |
| May 2022 | SAVP-D | Operating room 01 | Cabinet | CP+SS |
|  |  |  | Oxygen balloon | SS |
|  |  |  | Stainless steel supporting tray | CP+SS |
|  |  |  | Anaesthetic device buttons | SS |
|  |  |  | Anaesthetic device | CP+SS |
|  |  |  | Blanket | CP+SS |
|  |  |  | Thermal blanket | SS |
|  |  |  | Incubator table | CP+SS |
|  |  | Operating room 02 | Operating table - Head | CP+SS |
|  |  |  | Operating table - Toes | CP+SS |
|  |  |  | X-ray keyboard | SS |
|  |  |  | X-ray table | CP+SS |
|  |  | Cat ward | Stainless steel supporting tray | CP+SS |
|  |  |  | Blanket | CP+ SS |
|  |  |  | Big cage | CP+SS |
|  |  |  | Small cage | CP+SS |
|  |  | Waiting room | Weight scale | CP+SS |
|  |  | Wash area | Grids washing table | CP+SS |
| February 2023 | SAVP-B | Operating room 01 | Glass wall operating table | CP+SS |
|  |  |  | Wall operating table | CP+SS |
|  |  |  | Stainless steel supporting tray | CP+SS |
|  |  |  | Stainless steel supporting tray (against the wall) | CP+SS |
|  |  |  | Table with support material (e.g. compresses) | CP+SS |
|  |  |  | Anaesthetic device | CP+SS |
|  |  |  | Wood shelf | CP+SS |
|  |  |  | Operating light handle 01 | SS |
|  |  |  | Operating light handle 02 | SS |
| February 2023 | SAVP-B | Operating room 01 | Computer keyboard | SS |
|  |  |  | Computer mouse | SS |
|  |  |  | Blankets | CP+SS |
|  |  |  | Oxygen balloon | SS |
|  |  | Operating room 02 | Door-side operating table | CP+SS |
|  |  |  | Wall -side operating table | CP+SS |
|  |  |  | Thermal blanket | CP+SS |
|  |  |  | Anaesthetic device | CP+SS |
|  |  |  | Computer desk | CP+SS |
|  |  |  | Stainless steel supporting tray | CP+SS |
|  |  |  | Operating light handle | SS |
|  |  |  | Anaesthetic device buttons | SS |
|  |  |  | Oxygen balloon | SS |
|  |  |  | Computer keyboard | SS |
|  |  |  | Computer mouse | SS |
|  |  |  | Infusion pump | SS |
|  |  |  | Drain | SS |
|  |  | Treatment area | Stainless steel supporting table | CP+SS |
|  |  |  | Table with washing grids | CP+SS |
|  |  |  | Hallway table | CP+SS |
|  |  |  | Computer desk | CP+SS |
|  |  |  | Computer keyboard | SS |
|  |  |  | Computer mouse | SS |
|  |  |  | Shearing blade | SS |
|  |  |  | Ward side table | SS |
|  |  |  | Tap | SS |
|  |  |  | Liquid soap dispenser | SS |
|  |  | Dog ward | Stainless steel supporting tray | SS |
|  |  |  | Grids cage | CP+SS |
|  |  |  | Weight scale | CP+SS |
|  |  |  | Empty cage 01 | CP+SS |
| February 2023 | SAVP-B | Dog ward | Empty cage 02 | CP+SS |
|  |  | Cat ward | Stainless steel supporting tray | CP+SS |
|  |  |  | Empty cage | CP+SS |
|  |  |  | Infusion pump | SS |
|  |  |  | Sink | SS |
|  |  | Isolation unit | Weight scale | CP+SS |
|  |  |  | Table with support material (e.g. syringes) | CP+SS |
|  |  |  | Empty cage 01 | CP+SS |
|  |  |  | Empty cage 02 | CP+SS |
|  |  |  | Empty cage mat | CP+SS |
|  |  |  | Sink grids | CP+SS |
|  |  |  | Tap | SS |
|  |  |  | Antiseptic solution dispenser | SS |
|  |  |  | Single cage | CP+SS |
| September 2023 | SAVP-E | Operating room | Operating table - Head | CP+SS |
|  |  |  | Operating table - Toes | CP+SS |
|  |  |  | Thermal blanket | CP+SS |
|  |  |  | Anaesthetic device | CP+SS |
|  |  |  | Stainless steel stool on the toes of operating table | CP+SS |
|  |  |  | Sink | SS |
|  |  |  | Operating light handle | SS |
|  |  |  | Sink countertop | CP+SS |
|  |  |  | Chlorohexidine dispenser | SS |
|  |  |  | Incubator | CP+SS |
|  |  |  | Incubator hatch | SS |
|  |  |  | Tap | SS |
|  |  |  | Detergent dispenser | SS |
|  |  |  | Internal valve anaesthetic device | SS |
|  |  |  | Internal valve tubes anaesthetic device | SS |
|  |  |  | Oxygen balloon | SS |
| September 2023 | SAVP-E | Operating room | Operating table handle | SS |
|  |  | Pre-operative area | Treatment table | CP+SS |
|  |  |  | Weight scale | CP+SS |
|  |  |  | Tap | SS |
|  |  |  | Computer keyboard | SS |
|  |  |  | Computer mouse | SS |
|  |  |  | Grids | SS |
|  |  |  | Catheter saline solution | SS |
|  |  |  | Shearing blade | SS |
|  |  | Dog ward | Large left cage | CP+SS |
|  |  |  | Large right cage | CP+SS |
|  |  |  | Small left cage | CP+SS |
|  |  | Cat ward | Cage handle | SS |
|  |  |  | Cage left top | CP+SS |
|  |  |  | Cage left down | CP+SS |
|  |  |  | Cage right down | CP+SS |
|  |  |  | Shearing blade | SS |
| November 2023 | SAVP-F | Operating room 01 | Operating table -Head | CP+SS |
|  |  |  | Operating table - Toes | CP+SS |
|  |  |  | Stainless steel supporting tray | CP+SS |
|  |  |  | Buttons anaesthetic device | SS |
|  |  |  | Oxygen balloon | SS |
|  |  |  | Countertop with alcohol | CP+S |
|  |  |  | Operating light handle | SS |
|  |  |  | Wood stool | CP+SS |
|  |  | Operating room 03 | Operating table -Head | CP+SS |
|  |  |  | Operating table -Toes | CP+SS |
|  |  |  | Operating light handle | SS |
|  |  |  | Endoscopy table -Head | CP+SS |
|  |  |  | Endoscope table -Toes | CP+SS |
|  |  |  | Siemens keyboard | SS |
| November 2023 | SAVP-F | Operating room 03 | Countertop with material | CP+SS |
|  |  | Dog ward | Treatment table with plastic mat | CP+SS |
|  |  |  | Small cage | CP+SS |
|  |  |  | Computer keyboard | SS |
|  |  |  | Black shearing blade | SS |
|  |  |  | Light handle 01 | SS |
|  |  |  | Light handle 02 | SS |
|  |  |  | Red shearing blade | SS |
|  |  |  | Weight scale | CP+SS |
|  |  | Cat ward | Double cage | CP+SS |
|  |  | Intensive care unit | Countertop | CP+SS |
|  |  | Wash room | Tap | SS |
|  |  |  | Sink | SS |
|  |  |  | Drain | SS |
| November 2023 | SAVP-G | Operating room 01 | Operating table - Head | CP+SS |
|  |  |  | Operating table - Toes | CP+SS |
|  |  |  | Blanket | CP+SS |
|  |  |  | Anaesthetic device | SS |
|  |  |  | Operating light handle | SS |
|  |  |  | Computer keyboard | SS |
|  |  |  | Oxygen balloon | SS |
|  |  | Recovery ward | Treatment table plastic mat | CP+SS |
|  |  |  | Small cage | CP+SS |
|  |  |  | Large cage | CP+SS |
|  |  |  | Plastic mat | CP+SS |
|  |  |  | Shearing blade | CP+SS |
|  |  | Dog ward | Treatment table plastic mat | CP+SS |
|  |  |  | Computer keyboard | SS |
|  |  |  | Tap | SS |
|  |  |  | Cage | CP+SS |
| November 2023 | SAVP-G | Dog ward | Treatment table grids | SS |
|  |  | Cat ward | Rough table top | CP+SS |
|  |  |  | Stainless steel supporting tray | CP+SS |
|  |  |  | Tap | SS |
|  |  |  | Cage | CP+SS |
|  |  | Isolation unit | Grids | CP+SS |
|  |  |  | Plastic mat on countertop | CP+SS |
|  |  |  | Cage | CP+SS |
|  |  |  | Tap | SS |

# Supplementary Table 2 – Environmental sampling sites in clinics H-N

| **Date of collection** | **Small animal veterinary practices** | **Areas** | **Specific areas** | **Sampling methods** |
| --- | --- | --- | --- | --- |
| May 2022 | SAVP-H | Operating room | Anaesthetic device buttons | SS |
|  |  |  | Oxygen balloon | SS |
|  |  |  | Stainless steel supporting tray | CP+SS |
|  |  |  | Bed | CP+SS |
|  |  |  | Wood stool | CP+SS |
|  |  |  | Operating Table – Window side | CP+SS |
|  |  |  | Thermal blanket | CP+SS |
|  |  |  | Operating Table – Door side | CP+SS |
|  |  | Treatment area | Weight scale | CP+SS |
|  |  |  | Black mat | CP+SS |
|  |  |  | Grids | CP+SS |
|  |  |  | Stainless steel supporting tray | CP+SS |
|  |  |  | Computer keyboard | SS |
|  |  | Isolation unit | Cage 01 | CP+SS |
|  |  |  | Cage 02 | CP+SS |
|  |  | Ultrasound room | Ultrasound keyboard | SS |
|  |  |  | Ultrasound screen | CP+SS |
|  |  |  | Ultrasound Table | SS |
|  |  | Waiting room | Weight Scale | CP+SS |
|  |  | Examination room | Examination Table | CP+SS |
| June 2022 | SAVP-I | Operating room | Operating table_Head | CP+SS |
|  |  |  | Operating table_Toes | CP+SS |
|  |  |  | Stainless steel supporting Tray | CP |
|  |  |  | Electric scalpel | SS |
|  |  |  | Operating light handle | SS |
|  |  |  | Vital signs monitor | SS |
|  |  |  | Medicine cabinet | CP+SS |
|  |  |  | Oxygen balloon | SS |
|  |  |  | Anaesthetic device buttons | SS |
|  |  |  | Door knob (inside the operating room) | SS |
|  |  |  | Door knob (corridor) | SS |
|  |  | Pre-operative/Treatment area | Fridge handle | SS |
| June 2022 | SAVP-I | Pre-operative/Treatment area | Tap | SS |
|  |  |  | Sink grids | CP+SS |
|  |  |  | Shearing blade 01 | CP+SS |
|  |  |  | Treatment table plastic mat | CP+SS |
|  |  |  | Microscope countertop | CP+SS |
|  |  |  | Handle cabinet 01 | SS |
|  |  |  | Handle cabinet 03 | CP |
|  |  |  | Handle cabinet 05 | CP |
|  |  |  | Door knob outside | SS |
|  |  |  | Brush of shearing blade | SS |
|  |  | Ward | Stainless steel supporting tray | CP+SS |
|  |  |  | Cage 01 | CP+SS |
|  |  |  | Cage 02 | CP+SS |
| July 2022 | SAVP-J | Operating room | Operating table - Head | CP+SS |
|  |  |  | Operating table - Toes | CP+SS |
|  |  | Examination/Treatment room | Weight scale | CP+SS |
|  |  |  | Stainless steel supporting tray 01 | CP+SS |
|  |  |  | Stainless steel supporting tray 2 | CP+SS |
|  |  |  | Shearing blade | CP+SS |
|  |  |  | Brush of shearing blade | CP+SS |
|  |  |  | Otoscope | SS |
|  |  |  | Macrometric/micrometric microscope | SS |
|  |  |  | Platinum microscope | SS |
|  |  |  | Microscope countertop | CP+SS |
|  |  |  | Computer keyboard | CP+SS |
|  |  |  | Computer mouse | SS |
|  |  |  | Desk | CP+SS |
|  |  |  | Door knob 01 | SS |
|  |  |  | Door knob 02 | SS |
|  |  |  | Handle cabinet 01 | SS |
|  |  |  | Handle cabinet 02 | SS |
|  |  |  | Fridge handle | SS |
| July 2022 | SAVP-J | Examination/Treatment room | Trash | CP+SS |
|  |  |  | Tap | SS |
| September 2022 | SAVP-K | Operating room | Anaesthetic device buttons | SS |
|  |  |  | Oxygen balloon | SS |
|  |  |  | Upper cabinet | SS |
|  |  |  | Door knob - Inside | SS |
|  |  |  | Shearing blade cleaning brush | CP+SS |
|  |  |  | Anaesthesia Tent - Inside | CP+SS |
|  |  |  | Thermal mat | CP+SS |
|  |  |  | Anaesthesia tent - Outside | CP+SS |
|  |  |  | Door knob - Exterior | SS |
|  |  |  | Operating table - Head | CP+SS |
|  |  |  | Operating table - Toes | CP+SS |
|  |  |  | Drawer handle | SS |
|  |  |  | Door knob (operating room to hallway) | SS |
|  |  |  | Blanket | CP+SS |
|  |  |  | Light handle | SS |
|  |  |  | White countertop | CP+SS |
|  |  |  | Stainless steel supporting tray | CP+SS |
|  |  | Pre-operative/Treatment area | Pre-operating knob - Inside | SS |
|  |  |  | Fridge handle | SS |
|  |  |  | Sink grids | SS |
|  |  |  | Tap | SS |
|  |  |  | Sink | SS |
|  |  |  | Microwave table | SS |
|  |  |  | Pre-operative area cabinet handle | SS |
|  |  |  | Pre-operative area knob - Outside | SS |
|  |  | Ward | Small cage | CP+SS |
|  |  |  | Outside knob | SS |
|  |  |  | Drawer handles | SS |
|  |  |  | Pink blanket | CP+SS |
| September 2022 | SAVP-K | Ward | Inside knob | SS |
|  |  |  | Large cage | CP+SS |
|  |  |  | Bench | CP+SS |
|  |  |  | Tap | SS |
|  |  |  | Shearing blade | SS |
| March 2023 | SAVP-K | Operating room | Operating table - Toes | CP+SS |
|  |  |  | Operating table - Head | CP+SS |
|  |  |  | Stainless steel supporting tray | CP+SS |
|  |  |  | Thermal blanket | CP+SS |
|  |  |  | Shearing blade | CP+SS |
|  |  |  | Anaesthetic device buttons | SS |
|  |  |  | Infusion pump | SS |
|  |  |  | Cabinet handle | SS |
|  |  |  | Operating table (Inside handle) | SS |
|  |  |  | Light switch | SS |
|  |  | Pre-operative/Treatment area | Grey plastic mat | CP+SS |
|  |  |  | Bathtub grids | CP+SS |
|  |  |  | Fluid appliance | SS |
|  |  |  | Cabinet handles | SS |
|  |  |  | Shearing blade | CP+SS |
|  |  | Ward | Cage | CP+SS |
|  |  |  | Supporting countertop | CP+SS |
|  |  |  | Cabinet handles | SS |
|  |  |  | Infusion pump | SS |
|  |  | Dog examination room | Sink | SS |
|  |  |  | Plastic mat table | CP+SS |
|  |  |  | Computer keyboard | SS |
|  |  |  | Weight scale | CP+SS |
|  |  |  | Cabinet handles | SS |
|  |  |  | Computer mouse | SS |
|  |  | Cat examination room | Plastic mat | CP+SS |
| March 2023 | SAVP-K | Cat examination room | Computer keyboard | SS |
|  |  |  | Computer mouse | SS |
|  |  |  | Weight scale | CP+SS |
|  |  |  | Cabinet handles | SS |
|  |  |  | Sink | SS |
|  |  | Exotic animals examination room | Sink | SS |
|  |  |  | Weight scale | CP+SS |
|  |  |  | Plastic mat | CP+SS |
|  |  |  | Computer keyboard | SS |
|  |  |  | Computer mouse | SS |
|  |  |  | Cabinet handles | SS |
|  |  |  | Round table - Lunch table | CP+SS |
|  |  | Ultrasound room | Ultrasound table | CP+SS |
|  |  |  | Ultrasound buttons | SS |
|  |  |  | Ultrasound keyboard | SS |
|  |  |  | Ultrasound mouse | SS |
|  |  | Waiting room | Weight scale | CP+SS |
| March 2023 | SAVP-I | Operating room | Operating table - Toes | CP+SS |
|  |  |  | Operating table - Head | CP+SS |
|  |  |  | Stainless steel supporting tray | CP+SS |
|  |  |  | Anaesthetic device buttons | SS |
|  |  |  | Light handle | SS |
|  |  |  | Mindray buttons | SS |
|  |  |  | Operating table protection | CP+SS |
|  |  |  | Shearing blade | CP+SS |
|  |  |  | Shearing blade cleaning brush | CP+SS |
|  |  |  | Clean countertop | CP+SS |
|  |  | Pre-operative/Treatment area | Plastic table mat | CP+SS |
|  |  |  | Bathtub grids | CP+SS |
|  |  |  | Shearing blade | CP+SS |
|  |  |  | Shearing blade cleaning brush | CP+SS |
| March 2023 | SAVP-I | Pre-operative/Treatment area | Outside knob | SS |
|  |  |  | Microscope countertop | CP+SS |
|  |  | Ward 01 | Cage upper left corner | CP+SS |
|  |  |  | Sink | CP+SS |
|  |  |  | Countertop | SS |
|  |  |  | Cabinet handles | SS |
|  |  | Ward 02 | Lower cage | CP+SS |
|  |  |  | Countertop | CP+SS |
|  |  |  | Sink | SS |
|  |  | Dog examination room | Examination table | CP+SS |
|  |  |  | Countertop | CP+SS |
|  |  |  | Computer keyboard | SS |
|  |  |  | Computer mouse | SS |
|  |  |  | Weight scale | CP+SS |
|  |  |  | Cabinet handles | SS |
|  |  |  | Sink | SS |
|  |  | Cat examination room | Examination table | CP+SS |
|  |  |  | Countertop | CP+SS |
|  |  |  | Computer keyboard | SS |
|  |  |  | Computer mouse | SS |
|  |  |  | Cabinet handles | SS |
|  |  |  | Sink | SS |
|  |  |  | Weight Scale | CP+SS |
|  |  | Exotic animals examination room | Examination table | CP+SS |
|  |  |  | Countertop | CP+SS |
|  |  |  | Computer keyboard | SS |
|  |  |  | Computer mouse | SS |
|  |  |  | Cabinet handles | SS |
|  |  |  | Sink | SS |
|  |  | Waiting room | Weight scale | CP+SS |
| March 2023 | SAVP-L | Operating room | Operating table -Toes | CP+SS |
| March 2023 | SAVP-L | Operating room | Operating table -Head | CP+SS |
|  |  |  | Stainless steel supporting tray | CP+SS |
|  |  | Ward | Infusion pump | SS |
|  |  |  | Large shearing blade | CP+SS |
|  |  |  | Small shearing blade | CP+SS |
|  |  |  | Anaesthetic device buttons | SS |
|  |  |  | Sink | SS |
|  |  |  | Cage 04 | CP+SS |
|  |  | Dog examination rom | Examination table | CP+SS |
|  |  |  | Computer keyboard | SS |
|  |  |  | Computer mouse | SS |
|  |  |  | Sink | SS |
|  |  | Cat examination room 01 | Examination table | CP+SS |
|  |  |  | Computer keyboard | SS |
|  |  |  | Computer mouse | SS |
|  |  |  | Sink | SS |
|  |  | Examination room 02 | Countertop | CP+SS |
|  |  |  | Computer keyboard | SS |
|  |  |  | Computer mouse | SS |
|  |  |  | Sink | SS |
|  |  |  | Weight scale | CP+SS |
|  |  | Examination room 03 | Weight scale | CP+SS |
|  |  |  | Computer keyboard | SS |
|  |  |  | Computer mouse | SS |
|  |  |  | Sink | SS |
|  |  |  | Countertop | CP+SS |
|  |  | Waiting room 01 | Weight scale | CP+SS |
|  |  | Waiting room 02 | Weight scale | CP+SS |
| March 2023 | SAVP-M | Operating room | Operating table - Toes | CP+SS |
|  |  |  | Operating table - Head | CP+SS |
|  |  |  | Stainless steel supporting tray | CP+SS |
| March 2023 | SAVP-M | Operating room | Large shearing blade | CP+SS |
|  |  |  | Small shearing blade | CP+SS |
|  |  |  | Thermal blanket | CP+SS |
|  |  |  | Cabinet handles | SS |
|  |  |  | Anaesthetic device buttons | SS |
|  |  |  | Light handle | SS |
|  |  |  | Clean countertop | CP+SS |
|  |  | Pre-operative area | Countertop | CP+SS |
|  |  |  | Sink | SS |
|  |  |  | Cabinet handles | SS |
|  |  | Ward | Cage 06 | CP+SS |
|  |  |  | Countertop | CP+SS |
|  |  |  | Cabinet handles | SS |
|  |  |  | Sink | SS |
|  |  | Examination room 01 | Examination table | CP+SS |
|  |  |  | Sink | SS |
|  |  |  | Computer keyboard | SS |
|  |  |  | Computer mouse | SS |
|  |  | Examination room 02 | Examination table | CP+SS |
|  |  |  | Weight scale | CP+SS |
|  |  |  | Sink | SS |
|  |  |  | Computer keyboard | SS |
|  |  |  | Computer mouse | SS |
|  |  |  | Cabinet handles | SS |
|  |  | Waiting room | Weight Scale | CP+SS |
| November 2023 | SAVP-N | Operating room 01 | Operating table - Head | CP+SS |
|  |  |  | Operating table - Toes | CP+SS |
|  |  |  | Anesthetic device | CP+SS |
|  |  |  | Operating light handle | SS |
|  |  |  | Oxygen balloon | SS |
|  |  |  | Thermal mat | CP+SS |
| November 2023 | SAVP-N | Operating room 02 | Operating table -Head | CP+SS |
|  |  |  | Operating table - Toes | CP+SS |
|  |  |  | Stainless steel supporting tray | CP+SS |
|  |  |  | Light handle | SS |
|  |  |  | Computer keyboard | SS |
|  |  |  | Anaesthetic device | SS |
|  |  | Treatment area | Treatment table | CP+SS |
|  |  |  | Shearing blade | CP+SS |
|  |  |  | Treatment table grids | SS |
|  |  |  | Light handle | SS |
|  |  |  | Disinfection dispenser | SS |
|  |  |  | Tap | SS |
|  |  | Recovery ward | Small cage 06 | CP+SS |
|  |  |  | Large cage 02 | CP+SS |
|  |  | Fomites | Veterinarian mobile phone | SS |
|  |  |  | Hand cloth | CP+SS |
|  |  |  | Practice’s mobile phone | SS |
|  |  |  | Keyboard personal computer | SS |

# Supplementary Table 3 – SNP matrix distance between *Pseudomonas aeruginosa* strains found on SAVPs I, K, and L

| SNP-distance | *P. aeruginosa* H07  (NCBI ASM2257023v1) | A6E4P2 | A5R4P1 | A6Np4P1 |
| --- | --- | --- | --- | --- |
| *P. aeruginosa* H07  (NCBI ASM2257023v1) | 0 | 56132 | 56049 | 56046 |
| A6E4P2 | 56132 | 0 | 14081 | 14078 |
| A5R4P1 | 56049 | 14081 | 0 | 3 |
| A6Np4P1 | 56046 | 14078 | 3 | 0 |

**Supplementary Table 4 – SNP matrix distance between *Pseudomonas aeruginosa* strains found on different surfaces of SAVP-G.**

| SNP-distance | *P. aeruginosa* H02  (NCBI ASM2257047v1) | ER2F8P2 | ER3C4P3 |
| --- | --- | --- | --- |
| *P. aeruginosa* H02  (NCBI ASM2257047v1) | 0 | 1738 | 1738 |
| ER2F8P2 | 1738 | 0 | 0 |
| ER3C4P3 | 1738 | 0 | 0 |

**Supplementary Table 5 – SNP matrix distance between *Pseudomonas juntendi* strains found on SAVP-G.**

| SNP-distance | *P. juntendi* K37  (NCBI ASM2526364v1 ) | ER4C8A1 | ER1C4P2a |
| --- | --- | --- | --- |
| *P. juntendi* K37 (NCBI ASM2526364v1 ) | 0 | 2098 | 2098 |
| ER4C8A1 | 2098 | 0 | 36 |
| ER1C4P2 | 2098 | 36 | 0 |

**Supplementary Table 6 – SNP matrix distance between *Stenotrophomonas maltophilia* strains found on different surfaces and team of SAVP-G.**

| SNP-distance | *S.maltophilia* NCTC10498 (NCBI ASM1138692v1) | ER3D8P2 | ER2F4P2 | ER3F8A1 |
| --- | --- | --- | --- | --- |
| *S.maltophilia* NCTC10498 (NCBI ASM1138692v1) | 0 | 52315 | 51413 | 52318 |
| ER3D8P2 | 52315 | 0 | 57170 | 5 |
| ER2F4P2 | 51413 | 57170 | 0 | 57173 |
| ER3F8A1 | 52318 | 5 | 57173 | 0 |

**Supplementary Table 7 – SNP matrix distance between *Stenotrophomonas maltophilia* strains found on different surfaces and team of SAVP-N.**

| SNP-distance | *S.maltophilia* SM15  (NCBI ASM2111719v1 ) | X3Hp4P1 | X4Hp4P2 | EX3I8A1 | EX2I4A1 | EX2C4P3 |
| --- | --- | --- | --- | --- | --- | --- |
| *S.maltophilia* SM15  (NCBI ASM2111719v1 ) | 0 | 848 | 844 | 849 | 849 | 57099 |
| X3Hp4P1 | 848 | 0 | 6 | 9 | 9 | 56684 |
| X4Hp4P2 | 844 | 6 | 0 | 7 | 7 | 56680 |
| EX3I8A1 | 849 | 9 | 7 | 0 | 10 | 56683 |
| EX2IAP1 | 849 | 9 | 7 | 10 | 0 | 56684 |
| EX2C4P3 | 57099 | 56684 | 56680 | 56683 | 56684 | 0 |

**
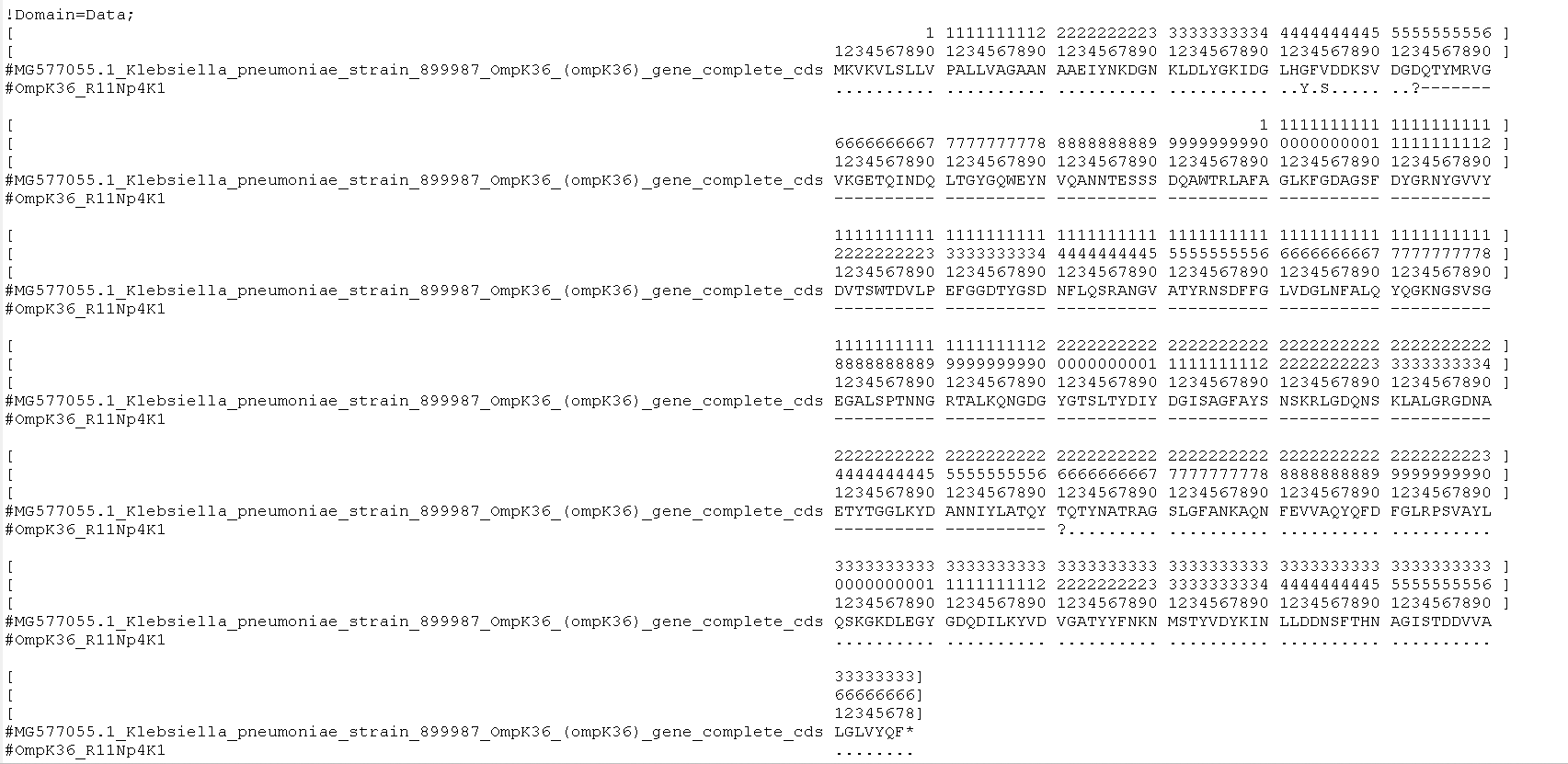
Supplementary Figure 1 – *Klebsiella pneumoniae* OmpK36 from R11Np4K1 and OmpK36 refence (GenBank: MG577055.1) from position 54-261 (highlighted in yellow)**
